# Purification and biochemical characterization of a secreted group IIA chicken intestinal phospholipase A_2_

**DOI:** 10.1186/1476-511X-10-27

**Published:** 2011-02-01

**Authors:** Aida Karray, Fakher Frikha, Yassine Ben Ali, Youssef Gargouri, Sofiane Bezzine

**Affiliations:** 1 Laboratoire de Biochimie et de Génie Enzymatique des Lipases, ENIS Route de Soukra, 3038 Sfax, University of Sfax -Tunisia

## Abstract

**Background:**

Secretory phospholipase A2 group IIA (IIA PLA2) is a protein shown to be highly expressed in the intestine of mammals. However, no study was reported in birds.

**Results:**

Chicken intestinal group IIA phospholipase A_2 _(ChPLA_2_-IIA) was obtained after an acidic treatment (pH.3.0), precipitation by ammonium sulphate, followed by sequential column chromatographies on Sephadex G-50 and mono-S ion exchanger. The enzyme was found to be a monomeric protein with a molecular mass of around 14 kDa. The purified enzyme showed a substrate preference for phosphatidylethanolamine and phosphatidylglycerol, and didn't hydrolyse phosphatidylcholine. Under optimal assay conditions, in the presence of 10 mM NaTDC and 10 mM CaCl_2, _a specific activity of 160 U.mg^-1 ^for purified ChPLA_2_-IIA was measured using egg yolk as substrate. The fifteen NH2-terminal amino acid residues of ChPLA_2_-IIA were sequenced and showed a close homology with known intestinal secreted phospholipases A_2_. The gene encoding the mature ChPLA_2_-IIA was cloned and sequenced. To further investigate structure-activity relationship, a 3D model of ChPLA_2_-IIA was built using the human intestinal phospholipase A_2 _structure as template.

**Conclusion:**

ChPLA2-IIA was purified to homogeneity using only two chromatographic colomns. Sequence analysis of the cloned cDNA indicates that the enzyme is highly basic with a pI of 9.0 and has a high degree of homology with mammalian intestinal PLA_2_-IIA.

## Background

Phospholipases A_2 _(PLA_2_s) hydrolyse the *sn-2 *bond of phospholipids resulting in the release of a fatty acid and lysophospholipid. Mammalian PLA_2_s are classified in three broad categories of secreted PLA_2 _(sPLA_2_) and cytosolic PLA_2_s which are either calcium dependent as to their catalytic activity, or calcium independent cytosolic PLA_2_s [1]. Secreted PLA_2_s are small molecular size proteins (14-19 kDa) with a rigid tertiary structure, having five to eight disulfide bonds that probably confer resistance to proteolysis and thermal denaturation [1,2] with a highly conserved catalytic site and a Ca^2+^-binding loop. These secreted PLA_2_s were first detected in snake venom pancreatic juice and in tissues [3]. Secreted PLA_2_s are also expressed in a number of cell types and present in various body fluids. They participate in the first line in antimicrobial defence of the body against bacteria and other pathogens. The extensive literature on sPLA_2_s in inflammatory diseases has been reviewed [4-11].

The gene of pancreatic PLA_2_-IB was first isolated in 1986 [12] followed in 1989 by the cloning of non-pancreatic PLA_2_-IIA from rheumatoid arthritic synovial fluid [13] and blood platelets [14]. Together with pancreatic PLA_2_-IB, the sPLA_2_-IIA are the best known and biochemically characterized enzymes. Novel sPLA_2 _were identified in the 90^th ^by screening nucleic acid data bases. Up to now, eleven sPLA_2_s have been cloned: IB, IIA, IIC, IID, IIE, IIF, III, V, X, and XIIA PLA_2_s together with the XIIB PLA_2_-like protein devoid of catalytic activity [1,15,16]. They express different catalytic and binding properties to natural phospholipids [17,18].

Earlier studies have localized sPLA_2_-IIA in the intestine [19,20] and in the synovial fluid of patients with rheumatoid arthritis as well as sPLA_2 _released from platelets [14,21]. The concentration levels of PLA_2_-IIA increase in sera of patients suffering from severe acute inflammatory diseases, such as sepsis and bacterial infections [22,23] and acute pancreatitis [24]. The sPLA_2_-IIA was originally localized in Paneth cells of the rat intestine [25,26] and later on in macrophages [27,28]. The two above mentioned cell types are both involved in the antibacterial response.

Later on it was demonstrated that the PLA_2_-IIA from human and mouse, with high activity on phosphatidylglycerol and bearing cationic properties (pI > 9.0), are highly bactericidal against gram positive bacteria [29-33] by perturbing the anionic bacterial cell wall [34]. The bacteria digested by the intestinal PLA_2 _do not necessarily have to be within the intestinal lumen. Some bacteria specifically invade the intestinal mucosa from the lamina propria, as it has been postulated to occur in Whipple's disease [35]. The human PLA_2_-IIA enzyme shows low affinity for zwitterionic interfaces, and in the absence of interfacial binding mammalian membrane hydrolysis is not possible [36].

With the growing interest in chicken diseases, such as avian influenza, we recently focused on bird sPLA_2 _to further gain some functional and pathological insights. Ben Bacha et al. [37] have biochemically characterized an active thermo stable PLA_2_-IB from ostrich pancreas (OPLA_2_). Another PLA_2_-IB was also purified from chicken pancreas and biochemically characterized [38]. It was therefore of interest to further study some biochemical and structural properties of avian non-pancreatic PLA_2 _to gain more insights into their mode of action on phospholipids and to compare their properties with those of mammalian PLA_2_. We report here, the purification and some biochemical properties of a secreted PLA_2 _from chicken intestine (ChPLA_2_-IIA). This work reports also the cloning of the corresponding bird PLA_2 _cDNA and the comparison of its deduced amino acid sequence with other known mammalian PLA_2_. A molecular 3D model of ChPLA_2_-IIA is also proposed to explain some biochemical differences of ChPLA_2_-IIA with other intestinal and pancreatic PLA_2_.

## Results and discussion

### Determination of phospholipase activity

**Figure 1:**
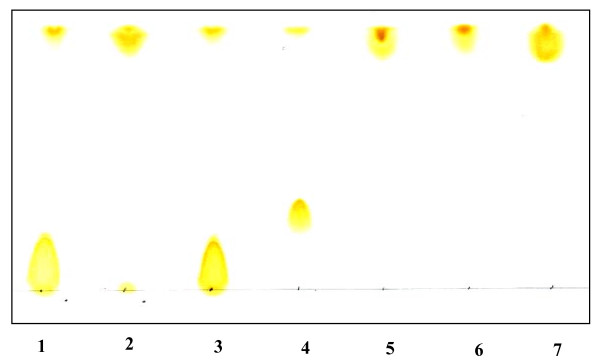
**Thin layer chromatography of PC and PE before or after incubation with ChPLA_2_-IIA or ChPLA_2_-IB**. After solvent migration the silica plate was exposed to iodine vapor to reveal the various spots. Lane (1), PC. Lane (2), PC incubated with PLA_2 _from chicken pancreas (PLA_2_-IB) and showing a complete hydrolysis of the PC. Lane (3), PC incubated with PLA_2 _from chicken intestine (PLA_2_-IIA). Lane (4), PE. Lane (5), PE incubated with PLA_2_-IB and showing a complete hydrolysis of the PE. Lane (6), PE incubated with PLA_2_-IIA and showing a complete hydrolysis of the PE. Lane (7), free fatty acid C 18:1.

The PLA_2 _activity was measured titrimetrically at pH 9.0 and at 40°C with a pH-stat, under the standard assay conditions as described previously [39], using egg yolk (0.5% W/V) as substrate, in 30 ml of 150 mM NaCl, 10 mM NaTDC and 10 mM CaCl_2_. The analysis of the purified egg yolk phospholipids by thin layer chromatography revealed the presence of two spots. The major one (90%) corresponds to the phosphatidylcholine (PC, or lecithin) and the second one (10%) phosphatidylethanolamine (PE). These two phospholipids were separated by adsorption chromatography on a silica gel column. Elution was performed using stepwise ratios of chloroform/methanol. We incubated the purified PC or PE samples with ChPLA_2_-IB or ChPLA_2_-IIA respectively and a thin layer chromatography was performed as shown in Figure 1. As expected, ChPLA_2_-IB hydrolyzes efficiently PC and PE since the spots of these two phospholipids totally disappeared and a spot of free fatty acid appeared. In contrast, ChPLA_2_-IIA didn't hydrolyze PC even after a long incubation period. Interestingly, PE was totally hydrolyzed by ChPLA_2_-IIA similarly to what observed with ChPLA_2_-IB. These analytical results clearly indicate that the hydrolytic activity of ChPLA_2_-IIA, measured with pH-stat using egg yolk as substrate, is mainly due to the hydrolysis of the PE fraction.

### Purification of ChPLA_2_-IIA

**Figure 2:**
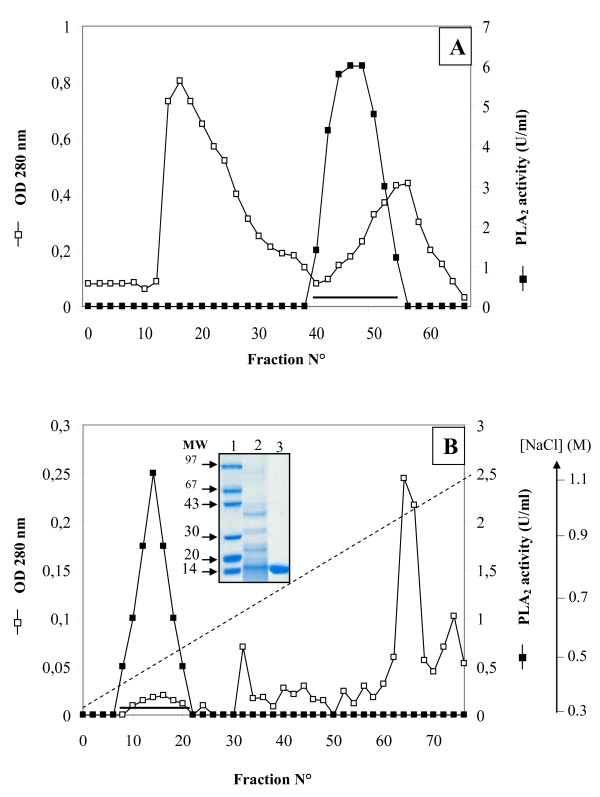
**Purification of ChPLA2-IIA**. **(A) **Gel filtration chromatography of intestinal ChPLA_2_-IIA on Sephadex G-50. The column (1.5 cm × 34 cm) equilibrated in 20 mM Tris-HCl buffer pH 8.0 containing 20 mM CaCl_2 _and 2 mM benzamidine. Elution was performed with the same buffer at a flow rate of 40 ml.h^-1 ^and 3 ml samples were collected. ChPLA_2_-IIA activity was measured as described in Material and methods section using egg yolk emulsion as substrate. The pooled fractions containing the PLA_2 _activity were indicated by horizontal line. **(B) **Mono-S Sepharose chromatography. The column (5 cm × 2 cm) was equilibrated with 20 mM Tris HCl buffer pH 8.0 containing 20 mM CaCl_2 _and 2 mM benzamidine; and then washed with the same buffer containing 0.3 M NaCl. Linear salt gradient (0.3 to 1 M NaCl, dotted line) was applied to the column; gradient chamber 75 ml; 2 ml fraction; flow rate, 40 ml/h. The pooled fractions containing the PLA_2 _activity were indicated by horizontal line. SDS-PAGE (15%) analysis of pure ChPLA_2_-IIA was inserted in Figure 2B. Lane 1, molecular mass markers (MM); Lane 2, 15 μg of proteins obtained after Sephadex G-50 chromatography; Lane 3, 15 μg of purified ChPLA_2_-IIA, obtained after Mono-S chromatography.

ChPLA_2_-IIA was purified from the intestine mucosa using an acidic treatment, ammonium sulfate precipitation, followed by two chromatography steps on Sephadex G-50, and Mono-S Sepharose according to the procedure described in Material and Methods (Figure 2). The main steps of the purification procedures and flow sheet are summarized in Table 1. The specific activity of pure ChPLA_2_-IIA reaches 160 U.mg^-1 ^when egg yolk was used as substrate, at pH 9.0 and at 40°C, in the presence of 10 mM NaTDC, and 10 mM CaCl_2_. The ChPLA_2_-IIA purification yield was about 10%. SDS-PAGE analysis of the purified ChPLA_2 _IIA eluted from the Mono-S column show that the enzyme exhibited one homogenous band corresponding to an apparent molecular mass of about 14 kDa (Figure 2). The calculated molecular mass, using the amino acid sequence is 13616Da. Furthermore, the calculated isoelectric pH was equal to pH 9.01.

**Table 1:** Flow sheet of chicken intestinal phospholipase A_2 _purification

Purification Step	**Total Activity (U)**^**a**^	**Total Protein (mg)**^**b**^	Yield (%)	Specific activity (U/mg)	Purification Factor
**Extraction** **(pH 8.5)**	250	5520	100	0.045	1

**Acidic treatment (pH3)**	123	205.5	49	0.6	13.33

**(NH_4_)_2_SO_4_** **Precipitation** **(60%)**	108	108	43	1	22.22

**Sephadex G-50**	95	6.41	38	14.8	328.88

**MonoS Sepharose**	25	0.156	10	160	3555

a) 1 Unit: μmole of fatty acid released per min using egg yolk as substrate in the presence of 10 mM NaTDC and in the presence of 10 mM CaCl_2_.

b) Proteins were estimated by Bradford method [30]. The experiments were conducted in triplicate.

### Enzymatic properties of the purified ChPLA_2_-IIA

#### Ca^2+ ^dependence

**Figure 3:**
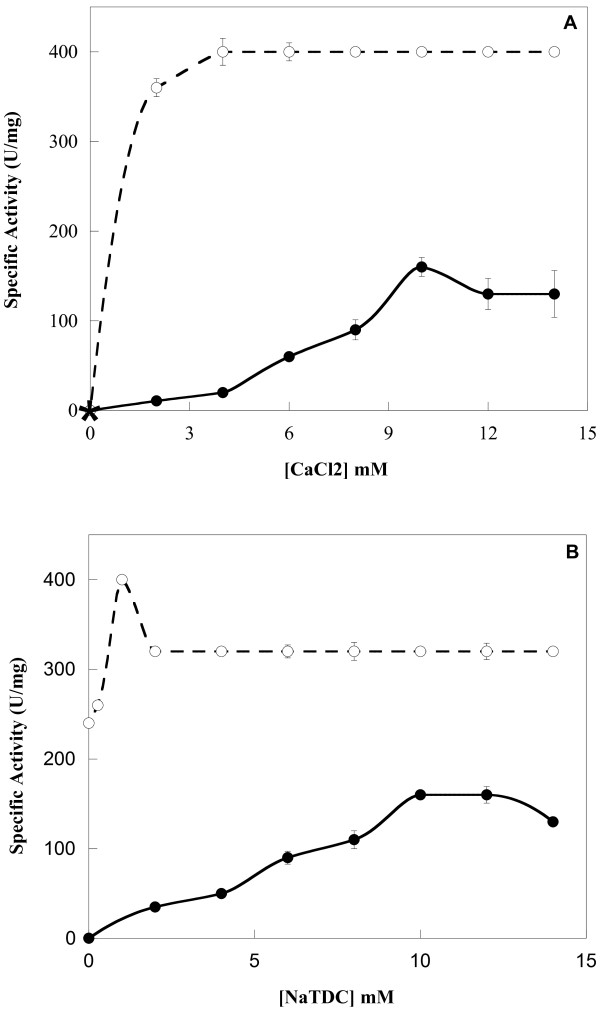
**Effect of Ca^2+ ^and NaTDC on ChPLA2-IB and ChPLA2-IIA activities**. **(A) **Effect of Ca^2+ ^concentration on ChPLA_2_-IIA (black circle) and ChPLA_2_-IB (white circle) activities. Enzyme activity was measured at various concentrations of Ca^2+ ^using egg yolk emulsion as substrate at pH 9.0 and at 40°C in the presence of 10 mM NaTDC. The star indicates the phospholipase activity measured in the absence of CaCl_2 _and in the presence of 10 mM EDTA or EGTA. **(B) **Effect of increasing concentration of bile salts (NaTDC) on ChPLA_2_-IIA (black circle) and ChPLA_2_-IB (white circle) activities. PLA_2 _activity was measured using egg yolk emulsion as substrate at pH 9.0 and at 40°C in the presence of 10 mM Ca^2+^.

It is well established that Ca^2+ ^is essential for both, catalysis and enzyme binding to the substrate [40-42]. In order to investigate the effect of Ca^2+ ^on ChPLA_2_-IIA activity, we studied the variation of hydrolysis rates of egg yolk phospholipids by pure ChPLA_2_-IIA in the presence of various Ca^2+ ^concentrations (Figure 3A). Our results showed that no PLA_2 _activity can be detected in the absence of Ca^2+ ^and in the presence of 10 mM EDTA or EGTA. In the absence of calcium chelators, the specific activity of purified ChPLA_2 _increases to reach 160 U.mg^-1 ^at 10 mM CaCl_2 _(Figure 3A).

These observations corroborate previous findings with porcine [20], rat [19], and human [14] intestinal PLA_2_. We previously reported that pancreatic chicken PLA_2_-IB requires only 4 mM of CaCl_2 _to reach its maximal activity (Figure 3A and [38]).

#### Bile salts dependence

Several studies have provided evidence that bile salts are tensioactive agents ensuring in their micellar form, the dispersion of the lipolytic products (of hydrolysis,) [43,44]. Along the same line, De Haas et al. reported that micellar forms of the substrate were hydrolysed at a much higher rate than substrates molecularly dispersed by PLA_2 _[45]. In this study, we measured the ChPLA_2_-IIA activity at pH 9.0 and at 40°C using egg yolk as substrate in the presence of increasing concentrations of bile salts. As shown in Figure 3B, NaTDC was required for ChPLA_2_-IIA activity. ChPLA_2_-IIA was poorly active at concentrations lower than 2 mM of NaTDC. This activity increases with increasing bile salts concentration and the maximal PLA_2 _activity was measured in the presence of 10 mM NaTDC. In contrast, we have presently shown, confirming our previous work, that pancreatic chicken PLA_2_-IB was found to be active in the absence of NaTDC and its optimal activity was found to be nearly independent of NATDC (Figure 3B and [38]. One has to recall that in the assay using egg yolk emulsion as substrate, the pancreatic PLA_2 _hydrolyses both PC and PE whereas the intestinal enzyme hydrolyses only PE.

#### Effect of temperature on ChPLA_2_-IIA activity and stability

**Figure 4:**
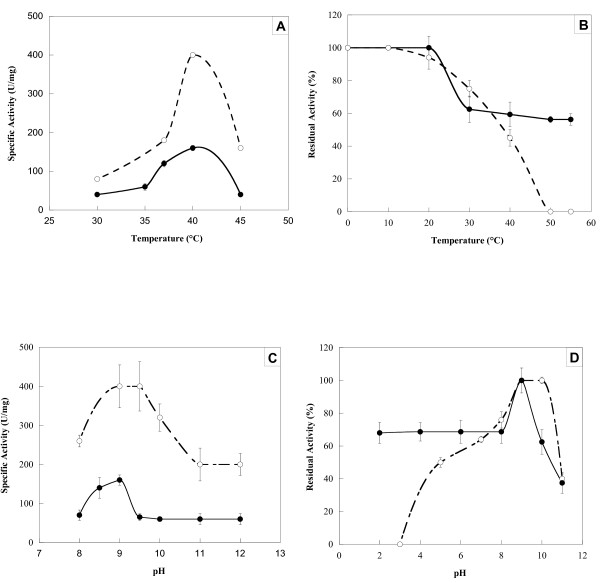
**Effect of temperature and pH on ChPLA2-IB and ChPLA2-IIA**. Effects of temperature **(A, B) **and pH **(C, D) **on ChPLA_2_-IIA (black circle) and ChPLA_2_-IB (white circle) activities **(A, C) **and stability **(B, D)**. PLA_2 _were tested for activity at various temperatures **(A) **and pH **(C) **as described in material and methods. To study the stability of PLA_2_, 1 mg.ml^-1 ^of each enzyme was incubated during 30 min at various temperatures **(B) **and pH **(D)**. Residual PLA_2 _activity was measured using egg yolk as substrate in the presence of 10 mM Ca^2+ ^and 10 mM NaTDC. For temperature stability studies, enzymes were incubated in 10 mM Tris (pH 8.0) and 10 mM CaCl_2_. For pH stability studies, Tris buffer was replaced by the appropriate buffer for the pH range.

Figure 4A shows that the maximal activity of ChPLA_2_-IIA was measured at 40°C using egg yolk as substrate in the presence of 10 mM Ca^2+ ^and 10 mM NaTDC. Unlike pancreatic ChPLA_2_-IB [38] which is totally inactivated at high temperature, the ChPLA_2_-IIA maintained about 60% of its activity after 15 min of incubation at 60°C (Figure 4B). Comparable results were obtained with mammalian intestinal PLA_2 _from various species which show a good stability at high temperature [46,47].

#### Effects of pH on ChPLA_2_-IIA activity and stability

As shown in Figure 4C, the maximal activity of ChPLA_2_-IIA was measured at pH 9.0 and at 40°C using egg yolk as substrate in the presence of 10 mM Ca^2+ ^and 10 mM NaTDC. Similar results were obtained with ChPLA_2_-IB and human intestinal PLA_2_-IIA. However, the purified ChPLA_2_-IIA was found to be stable between pH 3.0 and 10.0 (Figure 4D). In contrast to the ChPLA_2_-IB, which was found to lose its activity when incubated at pH lower than 5 [38], pure ChPLA_2_-IIA maintained about 50% of its activity after 15 min of incubation at pH 3.0 (see Figure 4D). It was also reported that porcine [20] rat [19] and human [14] intestinal PLA_2 _are stable at low pH values as compared to ChPLA_2_-IIA. Whereas, some pancreatic PLA2-IB are very acid and thermo stable [37,48]

#### N-terminal sequence of ChPLA_2_-IIA

The NH2 terminal sequencing of the ChPLA_2_-IIA allowed unambiguously the identification of the first fourteen residues of pure enzyme. Table 2 shows that the N-terminal sequence of ChPLA_2_-IIA has identity at 80% with mouse, 53% with human, and 53% with porcine PLA2-IIA respectively. Pure ChPLA2-IIA exhibits a high degree of homology with mouse intestinal PLA_2_-IIA.

**Table 2:** Alignment of the N-terminal amino-acid sequence of ChPLA_2_-IIA with mouse, human and porcine intestinal phospholipases. Identical aminoacids are in bold, and homologous aminoacids are in italic

Chicken:	N *I A *Q **F **G I **M I ***K *E K **T G K**	(present work)
**Mouse:**	N *I A *Q **F **G E **M I ***R *L K **T G K**	[19]

**Human:**	N *L V *N **F **H R **M I ***K *L T **T G K**	[14]

**Porcine:**	D *L L *N **F **R K **M I ***K *L K **T G K**	[20]

### Cloning and sequencing of the gene coding ChPLA_2_-IIA

**Figure 5:**
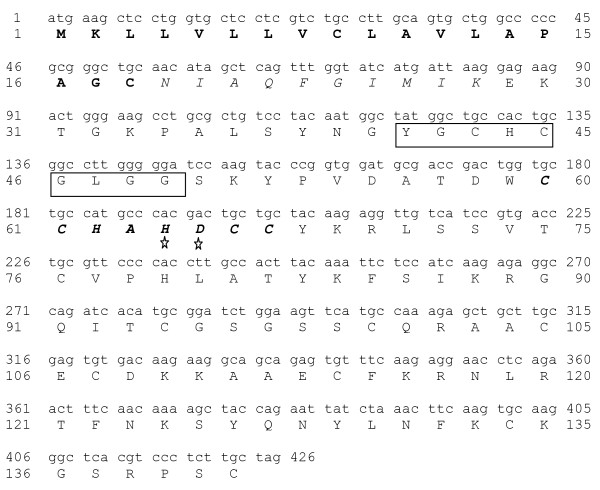
**Nucleotide sequence of the cDNA of ChPLA_2_-IIA and the deduced amino acid sequence**. Sequencing was performed in triplicate with three independent PCRs. The amino acid sequence obtained by N-terminal amino-acid sequence of the pure ChPLA_2_-IIA is shown in italic. M1-C18 in bold, signal peptide; Y41-G49 in square, Ca^2+ ^loop; and H64-D65 with stars, active site.

The cDNA encoding ChPLA_2_-IIA was selectively amplified by RT-PCR from total mRNA extracted from chicken intestine as described in material and methods. The corresponding DNA was ligated into pET 21a(+) vector and used for transformation into *E. coli *DH10B cells. Several clones are selected and some of them contained a recombinant plasmid with a 500 pb EcoRI/XhoI insert. The cDNA sequencing, confirmed that the PCR product corresponds to the gene encoding for the mature ChPLA_2_-IIA (Figure 5). The deduced polypeptide sequence of ChPLA_2_-IIA, corresponding to the mature protein, comprises 123 amino acids. ChPLA_2_-IIA shares 45% of identity with human PLA_2_-IIA and 51% of identity with mouse PLA_2_-IIA. Residues of the catalytic diad are conserved in ChPLA_2_-IIA. The 14 cystein residues involved in disulfide bridges in all known PLA_2 _IIA are also conserved in ChPLA2-IIA suggesting also the presence of 7 disulfide bridges in its 3D structure.

### Homology modelling

To provide an insight into the biochemical properties, the structure model of the ChPLA_2_-IIA was built, using the 3D structure of the hPLA_2 _(PDB code: 1N28) as template. These two proteins shares 45% amino acid identity. The model of the ChPLA_2_-IIA was then subjected to molecular mechanics optimization using CHARMM27 force-field. Energy minimization (geometry optimization) was performed until the gradient of 0.01 kcal/(Å.mol) was reached. The RMS deviations involving α-carbons between the initial and the optimized models was 0.95 Å. The Ramachandran plot statistics of the final ChPLA_2_-IIA model and of the hPLA_2_, determined using the PROCHECK program, showed that 97.2%, and 100% of the residues were either in the most favored or in the additional allowed regions, respectively.

### Overall 3D structure model of ChPLA_2_

**Figure 6:**
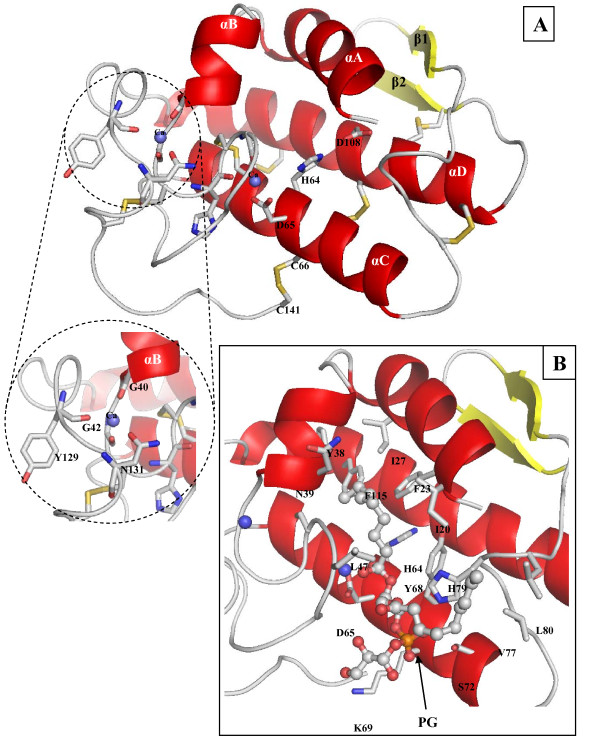
**3D modelling structure of ChPLA2-IIA**. **(A) **Cartoon representation the 3D model of the mature ChPLA_2_-IIA. Secondary structure elements labels are indicated. α-helices and β-strands are colored in red and yellow, respectively. The catalytic network and the Ca^2+^-binding residues are indicated and shown as sticks. The disulfide-bridges are shown in yellow. **(B) **Cartoon representation of the ChPLA_2_-IIA structure showing amino acids (represented by sticks) interacting with the substrate (PG). The substrate is indicated as a stick representation. The Ca^2+ ^ions are represented by a blue spheres. This figure was generated using the PYMOL software.

As shown in Figure 6, the ChPLA_2_-IIA has a globular shape with an α/β hydrolase fold stabilized by seven disulfide bridges similar to those present in all group IIA sPLA2. The core structure consists of four α-helices (αA: Ile2-Thr13; αB: Ala17-Gly22, αC: Ala38-Ser55 and αD: Ser81-Thr103) and two short anti-parallel β-strands (β1 : Phe67-Lys70 and β2 : Gln73-Cys76) located between helix αC and αD. The catalytic Ca^2+ ^ion is coordinated via oxygen atoms of His26 and Gly28 which belong to the Ca^2+^-binding loop and the two oxygen atoms, Oδ1 and Oδ2 of Asp47. The ChPLA_2_-IIA model showed that (O) Gly22, (O) Gly24, (O) Tyr111 and (Oδ1) Asn113 are located at an interaction distance of 2.2 Å with a second Ca^2+ ^ion. However these residues correspond in ChPLA_2_-IB to Asp24, Gly26, Leu118 and Lys120, respectively. No fourth amino acid was found in a favorable position to interact with this ion in ChPLA_2_-IB due to the substitution of Asn113 (Oδ1) (in ChPLA_2_-IIA) by Lys120 (in ChPLA_2_-IB). This second Ca^2+ ^ion may help the stabilization of the C-terminal (Tyr111 and Asn113) with the helix αB (Gly22, and Gly24) and furthermore the Ca^2+^-binding loop.

Although ChPLA_2_-IIA and ChPLA_2_-IB share seven disulfide bridges, only six of them are structurally conserved. In ChPLA_2_-IB, the disulfide bridge between Cys11 and Cys77 may stabilize the N-terminal α-helix αA with one of the two short strands of an anti-parallel β-sheet (β-wing) (β2). However, in ChPLA_2_-IIA, one new disulfide bridge is formed between Cys48 (belong to helix αC) and C-terminal Cys123.

To get more insight on the stabilization effect of the second calcium ion, molecular dynamics simulations were carried out (1 ns) at 330°K for the minimized models of the ChPLA_2_-IIA in the presence and the absence of this ion. The RMSD value for the backbone atoms was used to understand the response behavior. The 2D-RMSD plot, where the root mean square deviation of every conformation to all others of a simulation is shown, demonstrated that the conformational space sampled by ChPLA_2_-IIA in the simulations was larger in the absence of Ca^2+ ^ion (Figure 6).

### Docking

The total accessible surface area of the ChPLA_2_-IIA and ChPLA_2_-IB were 6591 and 6522 Å^2^, respectively. The positive charged surface of the ChPLA_2_-IIA (876 Å^2^, 12.6%) was higher than that of ChPLA_2_-IB (440 Å^2^, 6.7%). Furthermore, the negative charged surfaces of the ChPLA_2_-IIA and ChPLA_2_-IB were 69 Å^2^, (1%) and 399 Å^2^, (6.1%), respectively.

Analysis of PLA_2 _structures bound to a substrate can help in understanding the interaction mode of the substrate once located in the catalytic pocket.

The docking of the substrate (PC, PE or PG) to the ChPLA_2_-IIA and the ChPLA_2_-IB protein was performed using the Molegro Virtual Docker v.4.0.2 software, respectively (data not shown). According to the protein-substrate complexes, 13 or 12 residues are in van der vaals contact with the substrate in the case the ChPLA_2_-IIA and ChPLA_2_-IB, respectively. In the case of the ChPLA_2_-IIA, the total interaction energy between the protein and the target substrate is -134.8, -128.4 and -137,9 kJ/mol for PC, PE or PG respectively. In the case of the ChPLA_2_-IB, the total interaction energy is -183.8, -142.3 and -192.2 kJ/mol for PC, PE or PG respectively. These above mentioned interaction energy may help to explain the lack of catalytic activity of the ChPLA_2_-IIA on PC as substrate.

## Materials and methods

### Materials

Benzamidine was from Fluka (Buchs, Switzerland), bovine serum albumine (BSA), anhydrous magnesium sulfate, anhydrous sodium sulfate, potassium chloride, sodium chloride, taurodeoxycholic acid sodium salt (NaTDC) and PC were purchased from Sigma Chemical (St. Louis, MO, USA); acrylamide and bis-acrylamide electrophoresis grade were from BDH (Poole, UK), marker proteins and supports of chromatography used for phospholipase purification: Sephadex G-50, MonoS sepharose, were from Pharmacia (Uppsala, Sweden); protein sequencer Procise 492 equipped with 140 C HPLC system provided from Applied Biosystems (Roissy, France); pH-stat was from Metrohm (Herisau, Switzerland).

All enzymes and reagents used in DNA manipulations were from Promega and Invitrogen (Paris, France). Oligonuclotides were synthesized by Invitrogen. E. coli strain DH10B was used as cloning host for the gene part encoding the mature phospholipase. PCR products were purified using the Wizard PCR Preps DNA purification System (Promega).

Thin-layer Silica Gel 60 plates (10 × 20 cm from Merck) were used for the separation of lipids. Lipid standards (α-L-oleic acid C 18:1) and silica gel 60 powders (70-230) were from Sigma-Aldrich (Steinheim, Germany). All solvents with HPLC grade were purchased from SDS (Peypin, France).

### Lipid extraction, separation and analysis

Ten micrograms of purified ChPLA_2_-IB and purified ChPLA_2_-IIA were incubated respectively with 25 μM of pure phosphatidylcholine (PC) and phosphatidylethanol (PE) dispersion in 1 ml buffer (10 mM Tris HCl, 10 mM NaTDC and 10 mM CaCl_2_) at 37°C for 10 minutes. Lipolysis was stopped by adding 200 μl of 1 M HCL and mixing vigorously with 5 ml of chloroform/methanol mixture (2:1, v/v) in a 15 ml glass tube with a Teflon-lined screw cap. Lipids were immediately extracted as follows: after separation phases, the lower organic phase was transferred to a 15-ml test tube and dried over anhydrous magnesium sulphate. Once MgSO_4 _had precipitated, the clear organic phase was removed by centrifugation and stored at -20°C before TLC analysis.

To separate lipid classes, 1 to 50 μl of lipid extracts or lipid standards at known concentrations were first spotted onto a thin-layer silica plate. The elution of the lipids was then performed in one step with a chloroform/methanol/water (65/25/4, v/v/v) solvent mixture. Following chromatography, the plates were dried at room temperature for 10 min then immediately placed in the iodine.

### Enzyme samples

The intestine from chicken was collected from a local slaughterhouse (Sfax, Tunisia). Immediately after slaughter, the most distal intestine was opened on a glass plate on ice. The content of each ileal segment was flushed twice with 100 ml of 0.15 M NaCl. Adherent mesentery was removed as completely as possible.

### Purification of ChPLA_2_-IIA

Fifty grams of chicken intestine mucosa were suspended in 50 ml of buffer A (0.01 M Tris-HCl pH 8, 0.15 M NaCl, 0.02 M CaCl_2 _and 4 mM Benzamidine), and ground mechanically twice for 30 s at room temperature using the Waring Blendor system. Then, the mixture was stirred in a cold room for 1 h and centrifuged during 30 min at 12.000 rpm. The total PLA_2 _intestine activity obtained was 250 U.

### Acidic treatment

To inactivate proteins from the pancreatic juice and secreted into the intestine, the supernatant was brought to pH 3.0 by adding 6 N HCl under gentle stirring at 0°C. Insoluble denatured proteins were removed by centrifugation during 30 min at 12.000 g and the clear supernatant was adjusted to pH 8.0 with 4 N NaOH. The recovery of PLA_2 _activity was of about 50%.

### Ammonium sulphate precipitation

The supernatant (55 ml, 123 U) was brought to 60% saturation with solid ammonium sulphate under stirring conditions and maintained during 45 min at 4°C. After centrifugation for 30 min at 12.000 g and at 4°C, the precipitated PLA_2 _was resuspended in 2 ml of buffer A containing 2 mM benzamidine. Insoluble material was removed by centrifugation for 10 min at 24.000 g. The recovery of PLA_2 _activity was about 45%.

### Filtration on Sephadex G-50

The sample containing PLA_2 _activity (4 ml, 108 U) was loaded on a column of sephadex G-50 (34 cm × 1.5 cm) equilibrated with buffer B (20 mM Tris-HCl, pH 8 and 20 mM CaCl_2_). Elution of proteins was performed with the same buffer at 40 ml.h^-1^. The fractions containing the PLA_2 _activity eluted between 1.5 and 1.8 void volumes were pooled (Figure 2A).

### Cation exchange chromatography

The pooled fractions of Sephadex G-50 column containing PLA_2 _activity were poured into a Mono-S column (5 cm × 2 cm) equilibrated with buffer B. Under these conditions, the enzyme is adsorbed to the anionic support and the column was washed with 60 ml of the same buffer containing 0.3 M NaCl. ChPLA_2_-IIA was eluted by a linear salt gradient (0.3-1 M) NaCl as shown in Figure 2B.

### Analytical methods

Protein concentration was determined as described by Bradford et al. [49] using BSA $\left(\overset{1\%}{\underset{1\text{cm}}{\text{E}}}=6.7\right)$ as reference.

Analytical polyacrylamide gel electrophoresis of proteins in the presence of sodium dodecyl sulfate (SDS-PAGE) was performed by the method of Laemmli [50]. The proteins were stained with Coomassie brilliant blue.

### Amino acid sequencing

The N-terminal sequence was determined by automated Edman's degradation, using an Applied Biosystems Protein Sequencer Procise 492 equipped with 140C HPLC system.

### Bacterial starins, plasmids and media

*E. coli *strain DH10B was used as cloning host for the gene part encoding for the mature PLA_2_. *E. coli *strain was grown in Luria-Bertani medium, supplemented with 100 μg.ml^-1 ^ampicilline whenever plasmid maintenance was required. The plasmid pET21a(+) (Novagen) was used as cloning vector.

### cDNA synthesis and amplification

Total mRNAs were isolated from chicken intestine using the single step guanidine isothiocyanate/phenol/chloroform isolation method as described by Chomczynski and Sacchi [51]. ChPLA_2_-IIA cDNA was obtained from total mRNAs by the reverse transcription procedure (Promega). First strand cDNAs were prepared using 10 μg of total mRNAs as template (heat-denaturated for 5 min at 70°C,) 200 U MMLV reverse transcriptase (Invitrogen), 20 pmol of each deoxynucleoside triphosphate, and 20 pmol of each primer: forward primer, 5'- GAA TTC ATG AAG CTC C TG GTG CTC CT C -3' and reverse primer, 5'- CT C GAG CTA GCA AGA GGG ACG TGA GCC -3'. The N-terminal primer was predicted, from the N-terminal sequence of the ChPLA_2_; however the C-terminal primer was deduced from the genome of *Gallus gallus *(GenBank accession number: XP 424364). Reverse transcription was carried out in a total reaction volume of 20 μl for 5 min at room temperature and 60 min at 42°C. The cDNA/RNA heteroduplex was then denaturated at 70°C for 15 min and cooled on ice.

### Cloning of the mature PLA_2 _gene

Amplification of the specific ChPLA_2_-IIA cDNA was carried out by PCR using the single strand cDNAs as template with the forward and reverse primers previously described. PCR was performed in a 0.2 ml Eppendorf tube with a Gene Amp^® ^PCR System 2700. The PCR mixture contained 20 pmol of both primers, 20 pmol of each deoxynucleoside triphosphate, 5 U pfx polymerase and polymerisation buffer in final volume of 100 μl. The single strand cDNAs were directly used as template. The thermal profile involved 35 cycles of denaturation at 94°C for 1 min, primer annealing at 60°C for 1 min, and extension at 72°C for 3 min. The PCR product (500 pb) was isolated and ligated into the EcoRI and XhoI linearised and dephosphorylated pET21a(+) vector, according to the manufacturer's protocol (Promega). Protoplasts of *E. coli *DH10B were transformed with the ligation mixture. The resulting recombinant plasmid was named pChPLA_2_. The presence of the appropriated insert was verified by restriction analysis. DNA sequences were elucidated by the dideoxynucleotide chain termination method according to a cycle sequencing protocol using thermosequenase (Amersham Pharmacia Biotech). The sequencing reactions were analysed with the DNA sequencer ABI PRISM 3100/3100-Avant Genetic Analyser (California, USA). It was performed three times, using the recombinant vector pChPLA_2 _as template with T7 promoter primer and the T7 reverse primer (Invitrogen).

### Software for infrastructure

The sequence alignment was performed with BioEdit Version 4.8.4 software. The Molecular Operating Environment 2008.10 (MOE) software was used for homology modelling, molecular dynamics and structures visualization. Models were stereochemically evaluated by the program PROCHECK. The Visualization was performed with PyMol version 0.99beta06. Figures were generated by PyMol program.

### Homology Modelling

The 3-D coordinates of the human group IIA secreted PLA_2 _(hPLA_2_) (PDB code: 1N28) was extracted from the Protein Data Bank http://www.rcsb.org. The hPLA_2 _structure was used as template to build a model of the ChPLA_2_-IIA by using the structure-modelling program Molecular Operating Environment 2008.10. The model was then subjected to molecular mechanics optimization using CHARMM27 force field, until a gradient of 0.01 kcal/(Å.mol) was reached.

### Docking

The 1,2-dioctylcarbamoylglycero-3-O-phosphatidylcholine (PC), 1,2-dioctylcarbamoylglycero-3-O-phosphatidylethanolamine (PE) and 1,2-dioctylcarbamoylglycero-3-O-phosphatidylglycerol (PG) were modelled by the Molecular Operating Environment 2008.10 (MOE) software using a *Simplified Molecular Input Line Entry Specification (SMILES)*. The Molegro Virtual Docker v.4.0.2 software was used for docking substrates, PC, PE or PG to the ChPLA_2_-IIA and the ChPLA_2_-IB protein, respectively.

The potential binding sites (also referred to as cavities or active sites) was being identified using the built-in cavity detection algorithm, molecular surface with 0.5 Å grid resolutions and 1.2 Å Probe size.

After preparation of the protein and the ligand, the docking was performed using MolDock Score function, MolDock SE search algorithm, 10 runs, energy minimization and optimize H-Bonds after docking, 2500 iterations and 500 steps Simplex Evolution to generate the best five poses scores which were visually analyzed. Only one of these poses correctly bind to the active site with a high score was used. The protein-substrate complex was then subjected to molecular mechanics optimization using CHARMM27 force field as described previously. The Score of the final protein-substrate complex was evaluated using the total interaction energy between the active site and the substrate.

### Statistical analysis

All the results in figures and text are the average of at least three replicate experiments. They were statistically analyzed with SPSS software (version 100), using the Duncan test performed after analysis of variance (ANOVA).

## Abbreviation

PLA_2_: phospholipase A2; sPLA_2_: secreted PLA_2_; ChPLA_2_-IB: Group IB chicken pancreatic PLA_2_; ChPLA_2_-IIA : Group IIA chicken intestinal PLA_2_; hPLA_2_-IIA: Group IIA human PLA_2_; NaTDC: taurodeoxycholic acid sodium salt; PC: phosphtidylcholine; PE: phosphatidylethanolamine; TLC: thin layer chromatography; ns: nanosecond. MMLV: Moloney murine leukemia virus.

## Competing interests

The authors declare that they have no competing interests.

## Authors' contributions

AK carried out all the studies, analyzed the data and drafted the manuscript. FF carried out modelling and structural analysis. YBA helped with the analysis of the data and to correct the manuscript. YG helped with the discussion of the data and the correction of the manuscript. SB participated in the study design and helped to draft the manuscript. All authors have read and approved the final manuscript.
